# Fluctuations in Fabaceae mitochondrial genome size and content are both ancient and recent

**DOI:** 10.1186/s12870-019-2064-8

**Published:** 2019-10-25

**Authors:** In-Su Choi, Erika N. Schwarz, Tracey A. Ruhlman, Mohammad A. Khiyami, Jamal S. M. Sabir, Nahid H. Hajarah, Mernan J. Sabir, Samar O. Rabah, Robert K. Jansen

**Affiliations:** 1 Department of Integrative Biology, University of Texas at Austin, Austin, TX 78712 USA; 2 Department of Biological Sciences, St. Edward’s University, Austin, TX 78704 USA; 3 King Abdulaziz City for Science and Technology (KACST), Riyadh, 11442 Saudi Arabia; 4 Centre of Excellence in Bionanoscience Research, Department of Biological Sciences, Faculty of Science, King Abdulaziz University, Jeddah, 21589 Saudi Arabia; 5 Department of Biological Sciences, Faculty of Science, King Abdulaziz University, Jeddah, 21589 Saudi Arabia

**Keywords:** Comparative genomics, Gene loss, Genome size, Intron size, Repeats

## Abstract

**Background:**

Organelle genome studies of Fabaceae, an economically and ecologically important plant family, have been biased towards the plastid genome (plastome). Thus far, less than 15 mitochondrial genome (mitogenome) sequences of Fabaceae have been published, all but four of which belong to the subfamily Papilionoideae, limiting the understanding of size variation and content across the family. To address this, four mitogenomes were sequenced and assembled from three different subfamilies (Cercidoideae, Detarioideae and Caesalpinioideae).

**Results:**

Phylogenetic analysis based on shared mitochondrial protein coding regions produced a fully resolved and well-supported phylogeny that was completely congruent with the plastome tree. Comparative analyses suggest that two kinds of mitogenome expansions have occurred in Fabaceae. Size expansion of four genera (*Tamarindus*, *Libidibia*, *Haematoxylum*, and *Leucaena*) in two subfamilies (Detarioideae and Caesalpinioideae) occurred in relatively deep nodes, and was mainly caused by intercellular gene transfer and/or interspecific horizontal gene transfer (HGT). The second, more recent expansion occurred in the Papilionoideae as a result of duplication of native mitochondrial sequences. Family-wide gene content analysis revealed 11 gene losses, four (*rps2*, *7*, *11* and *13*) of which occurred in the ancestor of Fabaceae. Losses of the remaining seven genes (*cox2*, *rpl2*, *rpl10*, *rps1*, *rps19*, *sdh3*, *sdh4*) were restricted to specific lineages or occurred independently in different clades. Introns of three genes (*cox2*, *ccmFc* and *rps10*) showed extensive lineage-specific length variation due to large sequence insertions and deletions. Shared DNA analysis among Fabaceae mitogenomes demonstrated a substantial decay of intergenic spacers and provided further insight into HGT between the mimosoid clade of Caesalpinioideae and the holoparasitic *Lophophytum* (Balanophoraceae).

**Conclusion:**

This study represents the most exhaustive analysis of Fabaceae mitogenomes so far, and extends the understanding the dynamic variation in size and gene/intron content. The four newly sequenced mitogenomes reported here expands the phylogenetic coverage to four subfamilies. The family has experienced multiple mitogenome size fluctuations in both ancient and recent times. The causes of these size variations are distinct in different lineages. Fabaceae mitogenomes experienced extensive size fluctuation by recruitment of exogenous DNA and duplication of native mitochondrial DNA.

## Background

Mitochondrial genomes (mitogenomes) of plants exhibit drastic variation in size, architecture, gene content and nucleotide substitution rate [1, 2]. Early estimates of mitogenome size, mainly based on renaturation kinetics and restriction fragment analysis, ranged from 200 kb to 2.4 Mb (12-fold) [3]. More recent estimates using complete mitogenome sequences expanded this range from the exceptionally small genome of *Viscum scurruloideum* at 66 kb [4] to the large multipartite genome of 11.3 Mb in *Silene conica* [5]. This extensive size variation has been attributed to both internal and external factors. One primary internal cause is proliferation of repetitive sequences to include hundreds to thousands of repeat motifs [6, 7]. This repetitive DNA is also the main cause of rearrangements that generate the multipartite structure of mitogenomes [8, 9]. The predominant external factor in mitogenome expansion involves the gain of native DNA from other genomic compartments and foreign DNA from different organisms [10].

The exchange of genetic material among different genomic compartments within a cell is referred to as intracellular gene transfer (IGT) [11, 12]. Events involving IGT recruit DNA from the nucleus and plastid or export of mitochondrial DNA to the nucleus or plastid. Sequences within mitogenomes that originate in the nuclear genome and plastid genome (plastome) are referred to as mitochondrial DNA of nuclear origin (MINC) and mitochondrial DNA of plastid origin (MIPT), respectively [13]. Typically, MINCs are detected by sequence similarity to genes or transposable elements (TEs) encoded in the nucleus [14, 15]. In angiosperms, the majority of MIPTs are derived through IGT events originating within the cell. However, some MIPTs have a more complex history including a combination of IGT and interspecific mitochondrion-to-mitochondrion horizontal gene transfer (HGT). For example, MIPTs have been identified that were transferred by IGT in one species and then transferred by HGT to another, unrelated species [16]. The active and complex DNA exchange capability of mitogenomes [17, 18] through a combination of direct and vector-mediated mechanisms is not limited to plants but also occurs in arthropods, nematodes, protozoa, bacteria, fungi and viruses [19]. Thus, many of the plant mitogenomes that have been explored represent mosaics of DNA that originated from various sources, both from within individual cells and via exchange with other species [20].

After the endosymbiotic origin of the mitogenome [21], IGT events to the nucleus were accompanied with ‘protomitochondrial’ genome size reduction in the majority of eukaryotes [22]. However, in plant mitogenomes there has been extensive IGT and inter-specific HGT [23] resulting in exceptional variation in size and structure. Nuclear copies of mitochondrial genes may be degraded over time but sometimes they acquire regulatory elements for expression and intracellular targeting signals that direct transport of the product back to the mitochondrion [24]. Activation and targeting of the transferred gene render the mitochondrial copy dispensable often resulting in mitochondrial gene loss, which has occurred for many ribosomal protein and succinate dehydrogenase genes [25]. One notable gene loss in Fabaceae is *cox2* [26]. Extensive sampling of the phaseoloid lineage demonstrated the existence of many different conditions, with some taxa having copies of *cox2* in both the nucleus and mitochondrion and others where the mitochondrial copy was lost [27].

Fabaceae (legumes) are an excellent family for comparative genomics [28]. Among angiosperm families with model species, Fabaceae is the largest, including six subfamilies (Cercidoideae, Detarioideae, Duparquetioideae, Dialioideae, Caesalpinioideae, Papilionoideae), 770 genera and 20,000 species [29]. This diversity also makes the family an ideal system for evolutionary studies. Early investigations of angiosperm mitogenome evolution using Southern hybridization [27, 30–32] revealed several gene losses and IGT of mitochondrial genes to the nuclear genome within Fabaceae. Complete mitogenome sequencing [33–42] characterized many phenomena including gene loss, IGT, HGT and considerable variation in mitogenome size. Furthermore, recent studies elucidated unprecedented massive horizontal transfer of mitochondrial DNA from the mimosoid clade of Caesalpinioideae to the holoparasitic *Lophophytum* (Balanophoraceae) [39, 42, 43]. However, these comparisons only included 11 complete Fabaceae mitogenomes, nine of which were from Papilionoideae.

Recent improvements in sequencing technology have continuously advanced the understanding of plastome [44, 45] and mitogenome [2, 46] evolution. However due to the complexity of the mitogenome this progress has been biased towards plastomes, which in most lineages are highly conserved [47]. Many of the mitogenomes explored to date contain a preponderance of repetitive DNA, promiscuous DNA of unknown origin, gene losses and drastic genome rearrangement. The paucity of mitogenome sequences has hindered the understanding of the patterns and causes of variation. Here, four mitochondrial genomes from three different Fabaceae subfamilies were sequenced and assembled, two species from early diverging subfamilies (Cercidoideae and Detarioideae) and two species from non-mimosoid Caesalpinioideae. This allowed an investigation of mitogenome evolution among Fabaceae subfamilies, including horizontal transfer of the mitogenome to the holoparasitic *Lophophytum*.

## Results

### Genome assembly and finishing

The initial assembly of mitochondrial reads produced one to four large contigs for *Cercis canadensis*, *Tamarindus indica*, *Libidibia coriaria* and *Haematoxylum brasiletto*. Finishing with polymerase chain reaction (PCR) and Sanger sequencing generated a single master chromosome for each species. Genome sizes varied from 348,530 to 631,094 bp and average coverage of each of mitogenome was 150X to 490X (Table 1 and Additional file 1: Table S1). Coverage using paired end, plastome-filtered reads was fairly even across each genome (Additional file 2: Figure S1a). Total single end reads were also mapped to each completed mitogenome to show distribution of MIPTs (Additional file 2: Figure S1b). Constant genome coverage, together with PCR and Sanger sequencing of the MIPTs confirmed that the plastome-filtered assembly method successfully discriminated reads from the plastome and MIPTs in the mitogenomes.

**Table 1:** Summary of mitogenome features of Fabaceae

Subfamily	Species	Genome size (bp)	Number of unique intact genes			Transposable elements		MIPTs		Single copy region		Repetitive region	
			Protein-coding	rRNA	tRNA	%	Kb	%	Kb	%	Kb	%	Kb
Cercidoideae	*Cercis canadensis*	348,530	35	3	18	4.7	16.2	1.6	5.7	94.7	330.2	5.3	18.3
Detarioideae	*Tamarindus indica*	607,282	34	3	18	4.2	25.6	0.4	2.3	83.5	507.1	16.5	100.1
Caesalpinioideae	*Libidibia coriaria*	601,574	37	3	19	4.9	29	0.6	3.7	85.2	512.3	14.8	89.2
Caesalpinioideae	*Haematoxylum brasiletto*	631,094	37	3	20	5.1	32.3	1.2	7.7	81	511.3	19	119.8
Caesalpinioideae	*Leucaena trichandra*	729,504	36	3	19	5.1	37	1.1	8	75.7	552.4	24.3	177.1
Papilionoideae	*Millettia pinnata*	425,718	32	3	18	4.4	18.5	0.6	2.6	84.2	361	15.2	64.7
Papilionoideae	*Glycine max*	402,558	32	3	17	3.5	14.2	0.7	3	80.6	325	19.4	77.9
Papilionoideae	*Vigna angularis*	404,466	31	3	15	3.9	16	0.1	0.6	95.3	385.5	4.7	19
Papilionoideae	*Vigna radiata*	401,262	31	3	15	3.6	14.4	0.3	1.2	96.7	388.2	3.3	13
Papilionoideae	*Lotus japonicus*	380,861	31	3	17	4.4	16.6	1.5	5.8	84	320	16	61
Papilionoideae	*Vicia faba*	588,000	32	3	19	4	23.3	0.7	4.1	39.4	231.6	60.6	356.3
Papilionoideae	*Medicago truncatula*	271,618	32	3	16	4.3	11.6	0.4	1.1	97.1	263.7	2.9	7.9

*bp* basepairs, *kb* kilobasepairs

### Comparison of mitogenome and plastome phylogenies

**Fig. 1:**
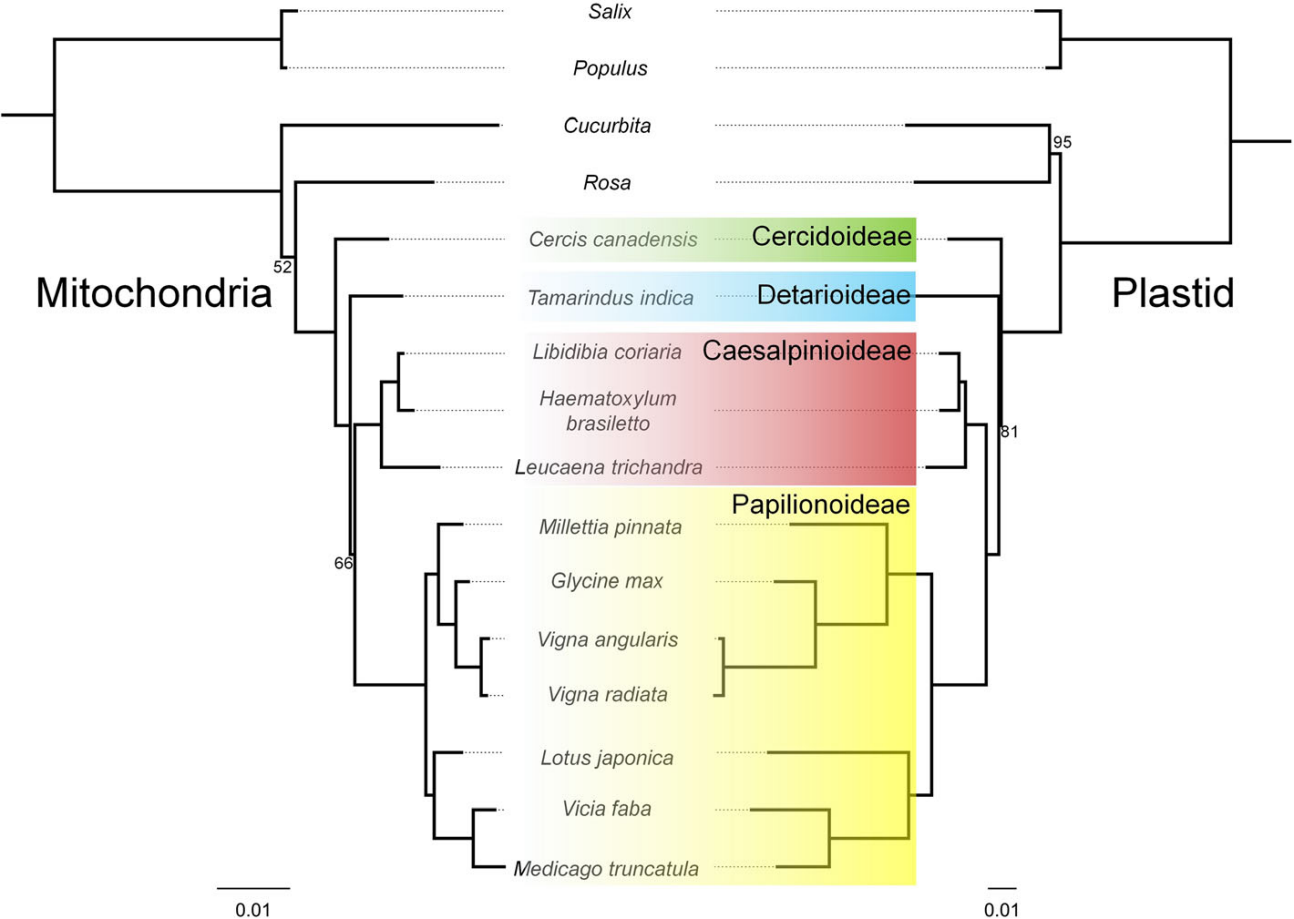
Maximum likelihood phylogenies of Fabaceae. Relationships were inferred employing mitogenome (26 protein coding genes) and plastome (68 protein coding genes) data (Additional file 1: Table S7). Bootstrap values < 100 are indicated at nodes. Gray dots extending from terminals indicate corresponding taxa in the two trees. NCBI accession numbers are listed in Additional file 1: Table S6. Scale indicates number of nucleotide substitutions per site

Maximum likelihood (ML) trees for Fabaceae were constructed using all shared protein coding genes from the mitogenome (26 CDS; 25,265 bp aligned length) and plastome (68 CDS; 52,497 bp aligned length) to assess congruence between the two organelle genomes (Fig. 1). Tree topologies of Fabaceae were congruent with high support (100% for all nodes except two nodes each in the mitogenome and plastome trees). Subfamily Cercidoideae diverged first followed by Detarioideae. Caesalpinioideae and Papilionoideae were each monophyletic and formed a well-supported clade that diverged next. Although there was no topological incongruence in Fabaceae between the two trees, there was substantial difference in branch lengths. The substitution ratio in mitogenomes versus plastomes from non-papilionoids was 1:3.7 and for papilionoids was 1:7.2. The mitogenome tree was used as a framework to evaluate changes in mitogenomic features across Fabaceae.

### Mitogenome composition and size variation

Genes, putative nuclear transposable elements (TEs), MIPTs and repeats were enumerated for 12 representative Fabaceae mitogenomes (Table 1). All genomes contained three rRNAs and the number of tRNAs ranged from 15 to 20. The number of protein coding genes varied from 31 to 37. Putative nuclear-derived TEs contributed 3.5—5.1% of the mitogenomes and this was closely correlated with genome size (Additional file 2: Figure S2a). The contribution of MIPTs to mitogenome size was minimal (0.1—1.6%), and less correlated to genome size than TEs (Additional file 2: Figure S2b).

Two kinds of repeat families, tandem and dispersed, were investigated in the 12 Fabaceae mitogenomes (Additional file 1: Table S2). The majority of repeats were dispersed and the total accumulative length of tandem repeats ranged from 0.1 to 1.8 kb among the species. Mitogenomes had a large and highly variable number of short (< 100 bp) dispersed repeats that were partly or wholly overlapping other short, intermediate (101—1000 bp) or large (> 1001 bp) repeats. The repeat pattern was extremely complex within each mitogenome. Accordingly, partly or wholly overlapping repeats were counted as a single repeat unit for calculating the total number of repeats.

**Fig. 2:**
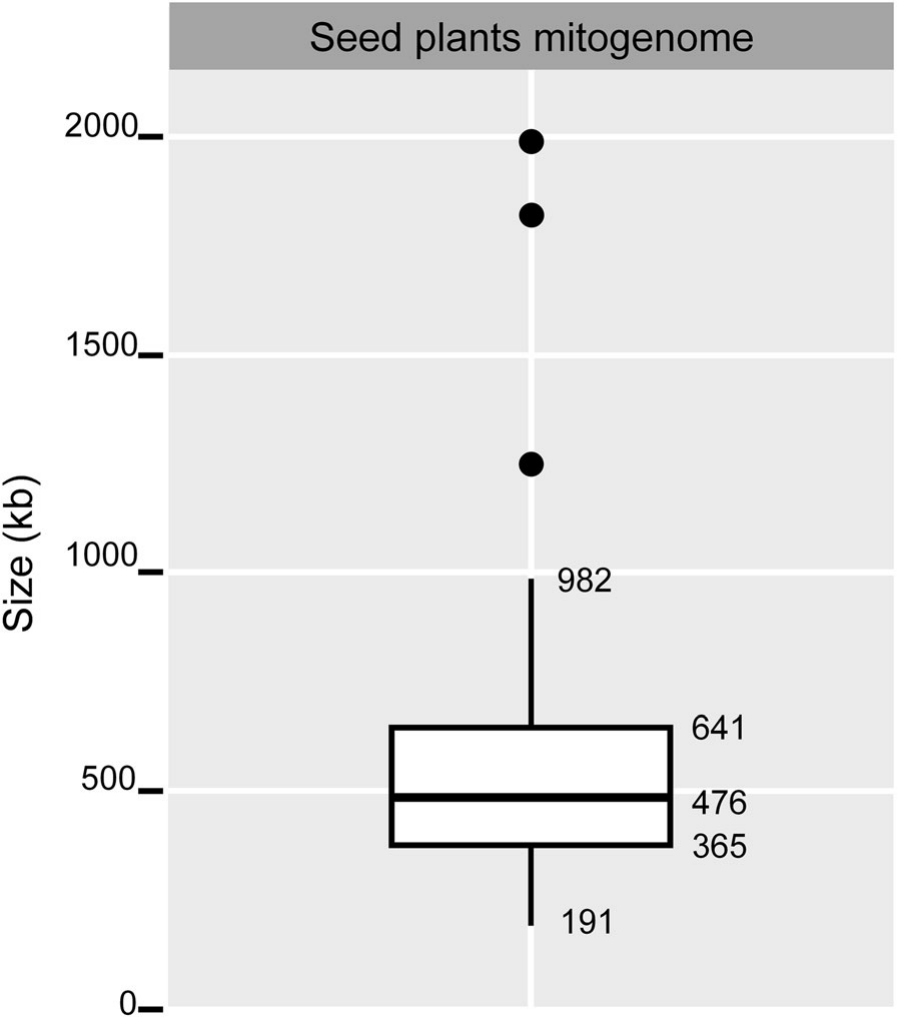
Size variation of mitochondrial genomes in seed plants. Data for seed plants was based on 106 available complete mitogenomes from NCBI (https://www.ncbi.nlm.nih.gov/genome/organelle/) accessed on Oct. 16, 2018. The mitogenomes without a single master chromosome are not included. The box shows interquartile range (IQR, middle 50%) and the horizontal line within the box is the mean. The black circles represent outliers that are beyond 1.5 IQR values. The whisker lines cover the range of data without outliers

**Fig. 3:**
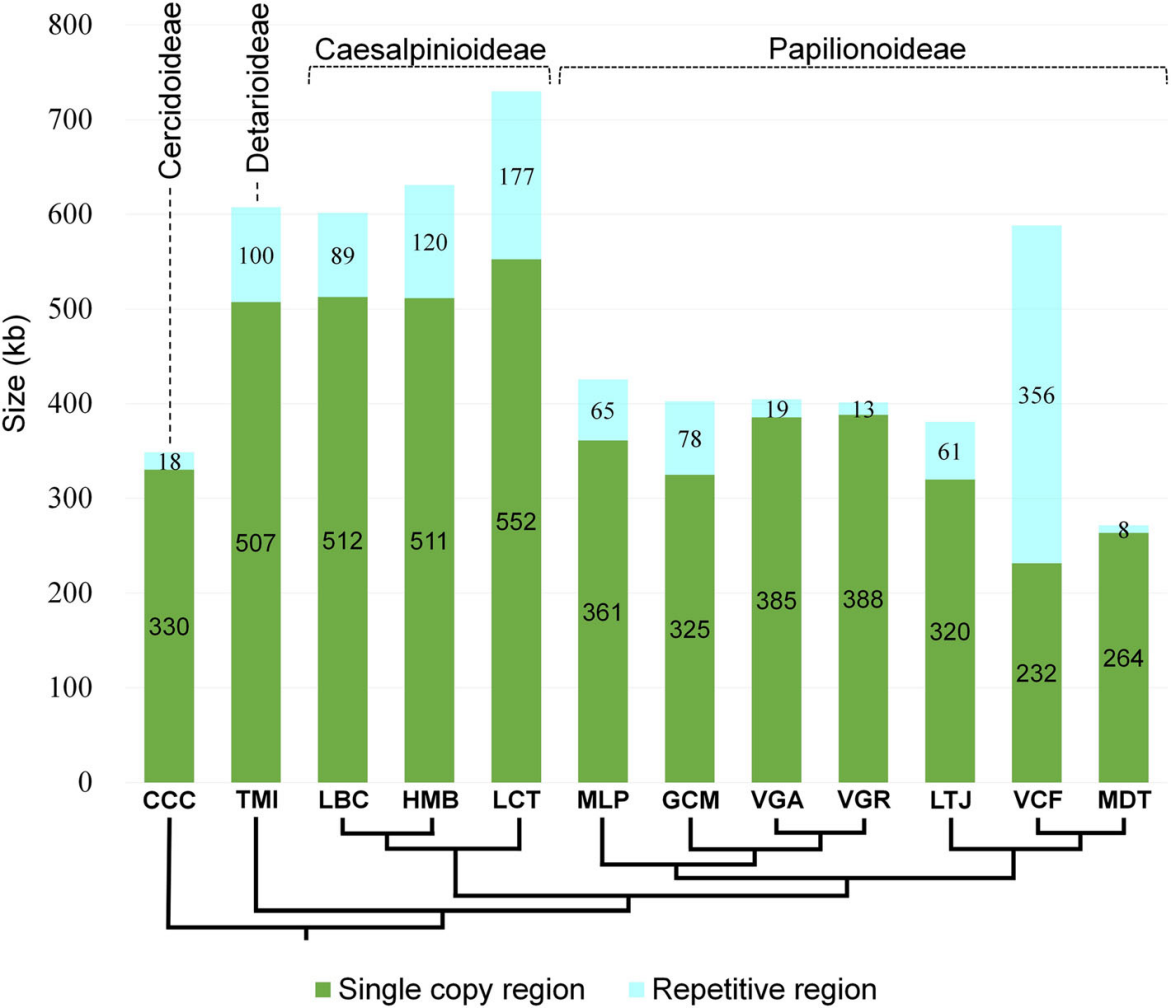
Single copy and repetitive DNA in Fabaceae mitogenomes. Single copy and repetitive regions were parsed and plotted on the phylogeny of Fabaceae from Fig. 1. Values within the histograms indicate the amount of sequence (in kb) contained in each region. CCC = *Cercis canadensis*, TMI = *Tamarindus indica*, LBC = *Libidibia coriaria*, HMB = *Haematoxylum brasiletto*, LCT = *Leucaena trichandra*, MLP = *Millettia pinnata*, GCM = *Glycine max*, VGA = *Vigna angularis*, VGR = *V. radiata*, LTJ = *Lotus japonicus*, VCF = *Vicia faba*, MDT = *Medicago truncatula*. Details of repeat content are in Additional file 1: Table S2

Mitogenome sizes varied considerably among Fabaceae, ranging from 271,618 bp (in *Medicago truncatula*) to 729,504 bp (in *Leucaena trichandra*) (ca. 2.7-fold; Table 1). The median size of seed plant mitogenomes (Fig. 2) was 476 kb, and overall variation ranged from 191 to 982 kb (roughly 5-fold, excluding outliers), and the interquartile range (IQR, middle 50%) ranged from 365 kb to 641 kb (roughly 2-fold). Based on the median size (476 kb) of mitogenomes of seed plants, Fabaceae mitogenomes formed two non-overlapping size groups, large (588—730 kb) and compact (272—425 kb). The size group with large mitogenomes consisted of four genera (*Tamarindus*, *Libidibia*, *Haematoxylum*, and *Leucaena*) in two subfamilies (Detarioideae and Caesalpinioideae) and *Vicia faba* (Papilionoideae, fava bean) (Table 1 and Fig. 3), while the compact size group included *Cercis* (Cercidoideae) and all other Papilionoideae species.

Mitogenome enlargement in *V. faba* was mainly caused by recent and rapid expansion of repeated sequences. Repetitive DNA constituted 60.6% (356.3 kb) of the genome (588.0 kb) (Table 1) but when all but one copy of large (> 1 kb) repeat sequences were excluded the genome size is 406.8 kb (Additional file 1: Table S2), which is similar to the other Papilionoideae mitogenomes (Fig. 3). Recent repeat expansion was evident in the total repeat accumulation in *V. faba* (Additional file 1: Table S2). Mitogenome enlargement in four genera (*Tamarindus*, *Libidibia*, *Haematoxylum*, and *Leucaena*) in two subfamilies (Detarioideae and Caesalpinioideae) cannot be attributed solely to recent repeat growth because the proportion of repeats was moderate (14.5—24.3%) and the amount of single copy sequence (500—552 kb) was very high compared to other subfamilies (Table 1 and Fig. 3).

### Evolution of mitogenome gene and intron content

**Fig. 4:**
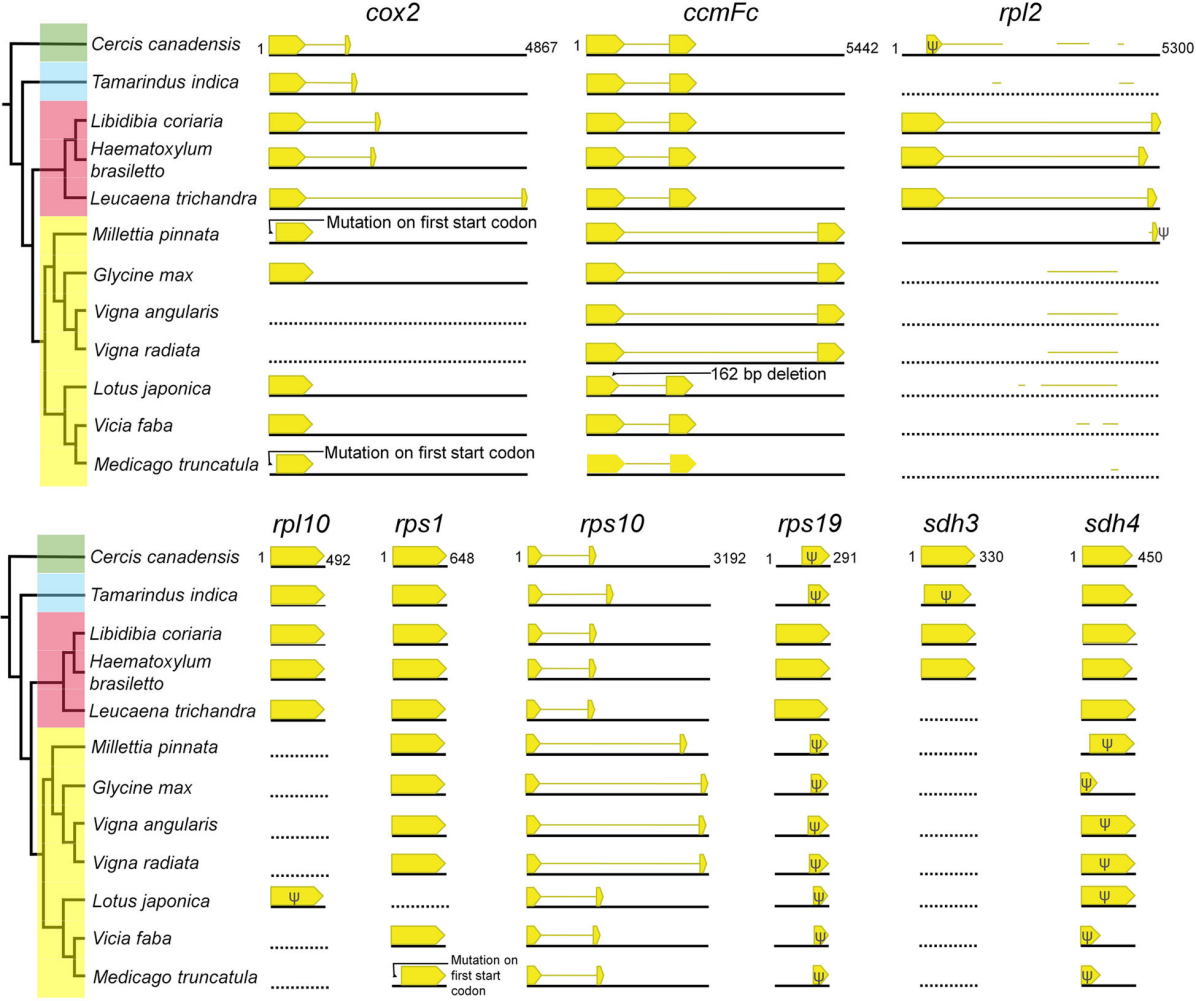
Highly variable mitochondrial genes in Fabaceae. Relationships among the taxa and corresponding subfamilies (green = Cercidoideae; blue = Detarioideae; red = Caesalpinioideae; yellow = Papilionoideae) are depicted in the cladogram on the left. The cladogram is drawn from the phylogenetic tree in Fig. 1. Yellow bars and lines indicate CDS and intron regions, respectively. Length of black line below each gene is based on longest copy of gene among the species, the value of which is given for *Cercis*. Genes without recognizable coding sequences are represented as dotted black lines. Putative pseudogenized or truncated genes are indicated with psi (Ψ). Sequence fragments (< 100 bp) are not presented. Position of sequence fragment was determined based on corresponding region of the longest gene. Intron and CDS length are proportional for the same gene but not between genes

The mitogenome phylogenetic tree in Fig. 1 was used to evaluate shared versus independent gene and intron losses within Fabaceae. Thirty of 41 protein coding genes were intact in all of Fabaceae mitogenomes (Additional file 2: Figure S3a). Among the remaining 11 genes, four ribosomal protein genes (*rps2*, *7*, *11*, and *13*) were lost (pseudogenized, truncated, or deleted) from all Fabaceae. The status of seven genes (*cox2*, *rpl2*, *rpl10*, *rps1*, *rps19*, *sdh3*, and *sdh4*) was variable in the family (Fig. 4). There was also a unique 162 bp in-frame deletion (54 amino acids) from *ccmFc* of *Lotus* relative to *Cercis*. All members of the subfamily Papilionoideae shared the losses of *rpl10* and *sdh4* although the amount of residual sequence differed across taxa. Losses of *cox2* and *rps1* were unique to the papilionoid genera *Vigna* and *Lotus*, respectively. Three genes, *rpl2*, *rps19* and *sdh3*, were lost multiple times among the subfamilies of Fabaceae. The *sdh3* coding sequence was lost three times, from *Tamarindus* (Detarioideae), *Leucaena* (Caesalpinioideae), and all sampled Papilionoideae. An intact *rpl2* and *rps19* were only present in Caesalpinioideae. ML analysis was performed on the sequences of these two genes and the resulting trees supported a Fabaceae origin (truncated in *Cercis* and intact in Caesalpinioideae) because in both cases Fabaceae formed a strongly supported monophyletic group within the rosid clade (92 and 89% bootstrap values, Additional file 2: Figure S4). These results indicated that Caesalpinioideae retained native copies of *rpl2* and *rps19* and the other subfamilies experienced multiple losses.

Extensive variation in the presence/absence and length of introns was detected in three genes *ccmFc* (953—4100 bp), *cox2* (0; 732—4080 bp), and *rps10* (842—2829 bp) (Fig. 4). Introns of *ccmFc* and *rps10* were greatly expanded in a monophyletic subset of Papilionoideae (*Millettia*, *Glycine* and *Vigna*). The loss of the *cox2* intron occurred in all Papilionoideae. Among non-papilionoids, the length of the *cox2* intron varied by species and was substantially elongated in *Leucaena*, a member of mimosoid clade of Caesalpinioideae. Multiple alignment revealed an additional 2.9 kb of sequence in the *Leucaena cox2* intron (Additional file 2: Figure S5). Using this sequence as a BLASTN query returned no significant match in the NCBI database. There was no strong match to known transposable elements from the CENSOR database. A BLAST search against the four mitogenomes completed in this study showed a strong match to an intergenic spacer (IGS) in *Haematoxylum* (position 15,062—17,424). Alignment of the additional *cox2* intron sequences of *Leucaena* and the corresponding IGS of *Haematoxylum* showed 94.9% sequence identity over 2.4 kb (Additional file 2: Figure S5). In the mitogenome of *Leucaena*, this additional *cox2* intron sequence was not present in the IGS region. The tandem repeat sequence (period size: 51, copy number: 4) was located the near 3′ end of the unique sequence but flanking repeat sequences were not identified. Caesalpinioideae have a noticeably long intron of *rpl2* (4017—4307 bp) (Fig. 4). However, it was uncertain if this intron was expanded in Caesalpinioideae due to a lack of reference mitogenomes from close relatives within the family. All other Fabaceae mitogenomes, including species lacking recognizable exon sequences of *rpl2*, had fragmented sequences similar to introns in Caesalpinioideae.

### Pairwise variation of shared DNAs and genetic distance

**Fig. 5:**
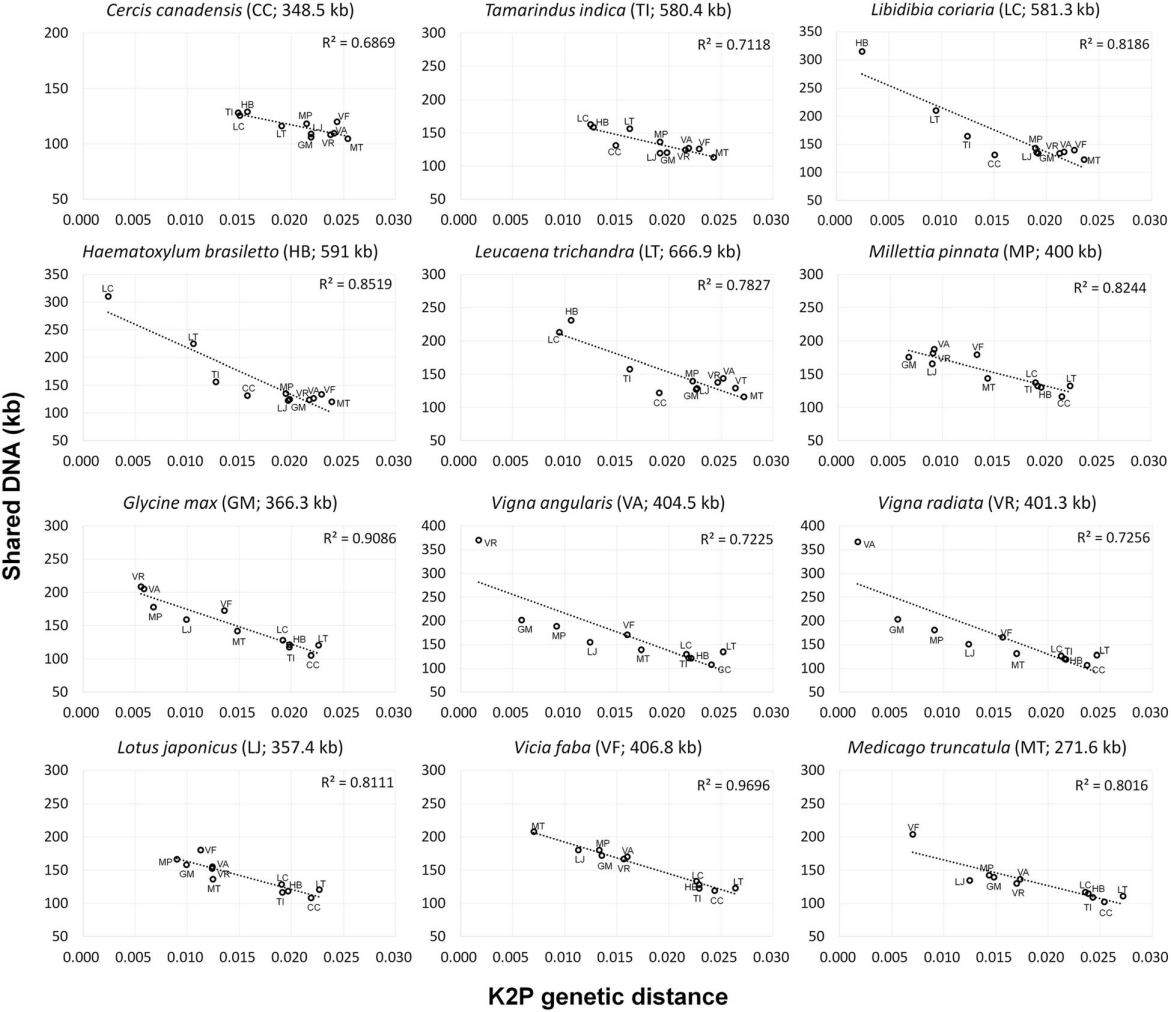
Relationship between shared DNA and K2P genetic distance in 12 representative Fabaceae mitogenomes. Taxon names on individual plots represent the query species and the dots within each represent the 11 subject Fabaceae species. Each subject species is represented by a two letter acronym and mitogenome size, excluding abundant large repeat copies (> 1 kb), in parentheses above each graph. Complete information on shared DNA and K2P genetic distance are in Additional file 1: Tables S3 and S4

Shared DNA content and Kimura 2-parameter (K2P) genetic distance was evaluated among Fabaceae mitogenomes (Additional file 1: Tables S3 and S4) and the correlation of these two parameters was tested. The analysis supported a strong negative correlation for each species (Fig. 5). Even closely related species with similar total genome size shared little DNA. For example, two Caesalpinioideae genera (*Libidibia* and *Haematoxylum*) with 0.24% divergence only shared about 50% of their mitogenome (Additional file 1: Tables S3 and S4). One example from Papilionoideae clearly demonstrated a rapid decrease of shared DNA in early stages of divergence. Intrageneric variation in *Vigna* (0.1% divergence) showed 92% shared mitogenome DNA while an intergeneric comparison between *V. angularis* and *Glycine* (0.59% divergence) showed ca. 50% shared DNA. Most inter-generic comparisons shared less than 50%. Across all comparisons at least 100 kb of DNA was shared, and this mainly comprised genes and their flanking regions.

### Shared DNA between Fabaceae and *Lophophytum*

**Fig. 6:**
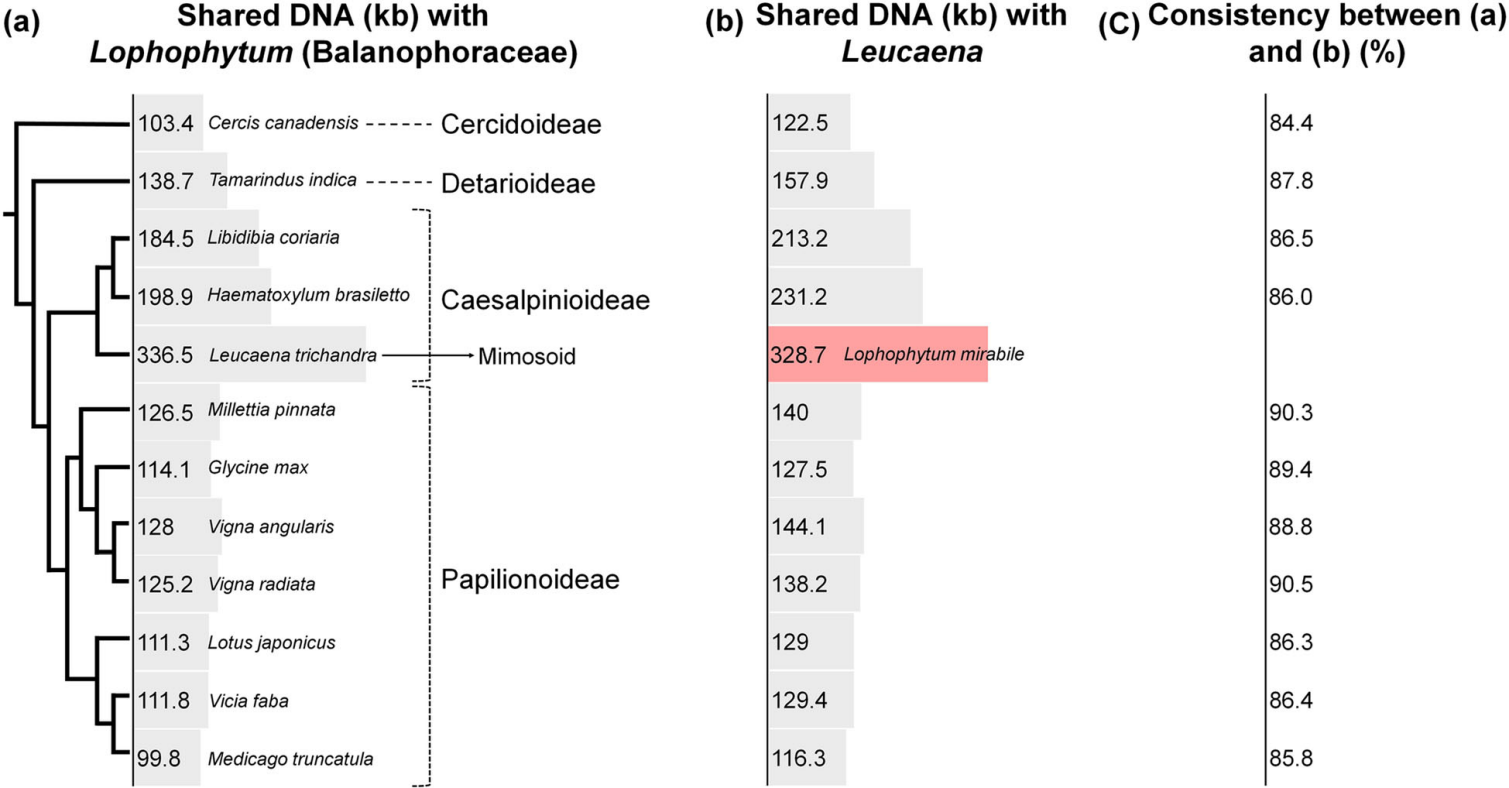
Shared DNA between Fabaceae and holoparasitic *Lophophytum* (Balanophoraceae) mitogenomes. (**a**) The mitogenome of *Lophophytum mirabile* was used to query a subject database comprising the 12 included Fabaceae. (**b**) The mitogenome of *Leucaena trichandra* was to query a subject database including the other 11 Fabaceae and *Lophophytum mirabile*. (**c**) Consistency in estimates of shared DNA between (**a**) and (**b**) were calculated. The cladogram is drawn from the phylogenetic tree in Fig. 1. Taxon names are indicated next to each shared DNA value. The taxon name for gray bar from (**a**) and (**b**) is identical, while the red bar indicates that *Lophophytum* replaced *L. trichandra* in the analysis

BLAST comparisons of the mitogenome of holoparasitic *Lophophytum* (Balanophoraceae) were performed with each of the 12 Fabaceae mitogenomes (Fig. 6a). *Leucaena* shared the greatest amount of *Lophophytum* DNA (336.5 kb). Within Papilionoideae, the amount of shared DNA varied from 99.8—126.5 kb. In non-papilionoids, there was a gradual increase in shared DNA that correlated with phylogenetic affinity to *Leucaena*. This pattern was very similar to analyses that used the mitogenome of *Leucaena* as a query sequence to other 11 Fabaceae species and *Lophophytum* (Fig. 6b). In this comparison the shared DNAs of *Leucaena* with *Lophophytum* was 328.7 kb. The consistency between these two analyses was the amount of shared DNA, most of which represented the ~ 100 kb of genes and conserved flanking regions (Fig. 6c).

## Discussion

### Causes of mitogenome size variation

Reduction of organelle genome size is observed in the majority of eukaryotic lineages and selective pressure towards genome compaction may provide a replicative advantage [22]. Within land plants, however, mitogenome size is generally expanded compared to its counterpart in most animals [23]. The mitogenomes of Fabaceae show two distinct patterns of size change (Table 1 and Fig. 3). Constraints on plant mitogenome size remain obscure, however, relaxed selection on genome size is one reasonable explanation for highly variable and large mitogenomes (Fig. 2). Considering the low copy number of mitogenomes per plant mitochondrion (frequently only one or a partial copy) [48], one source of selection, competition in replication among mitogenomes within mitochondria [49], may be reduced relative to animals. The somewhat stable genome size among the paraphyletic subset of Fabaceae [e.g. *Tamarindus* to *Haematoxylum*, *Millettia* to *Lotus* (Fig. 3 and Additional file 1: Table S2)] suggests that the integrity of mitogenomes has been sustained through inheritance and stoichiometric shifts to considerably different genome sizes is not common in the family.

It is evident that there have been several independent mitogenome size fluctuations in Fabaceae (Fig. 3). However, it is difficult to determine the number and timing of fluctuations because the current taxon sampling remains sparse considering the overall size and diversity of Fabaceae (770 genera and 20,000 species) [29]. It is noteworthy that there is a coincident pattern between mitogenome size fluctuation and nuclear chromosome evolution [50–52]. *Cercis*, a member of the earliest-diverging clade Cercidoideae with the second smallest mitogenome (Table 1), has a non-polyploid relic nuclear genome [52]. Whole genome duplications have occurred near the base of the other five subfamilies [50, 51]. The four genera *Tamarindus*, *Libidibia*, *Haematoxylum*, and *Leucaena* in subfamilies Detarioideae and Caesalpinioideae have enlarged mitogenomes. The recently published mitogenome of *Styphnolobium*, an early diverging genus of Papilionoideae, also has a larger size at 484,916 bp compared to other papilionoid genera [40]. These observations suggest that substantial mitogenome size fluctuations, may have been accompanied by nuclear chromosome evolution in deep nodes of Fabaceae. Expanded taxon sampling of both mitogenomes and nuclear genomes across the family are needed to confirm this pattern.

Deciphering the origin of promiscuous DNA within mitogenomes is challenged by the paucity of reference genomes and the fast-evolving nature of nuclear and mitogenome intergenic sequences [10, 53]. Mitogenome enlargement in the four genera *Tamarindus*, *Libidibia*, *Haematoxylum* and *Leucaena* in Detarioideae and Caesalpinioideae may be a consequence of integration of promiscuous DNA of unknown origin (Fig. 3). The lack of a reference nuclear genome and the fact that the additional promiscuous sequences are not conserved among the species (Fig. 6) hinders tracing the origin of the sequences. Phylogenetic analysis using mitochondrial coding sequences did not show strong signal of interspecific HGT (Fig. 1 and Additional file 2: Figure S4) and the contribution of MIPTs is very low and not strongly correlated to genome size (Table 1 and Additional file 2: Figure S2b). These lines of evidence suggest it is unlikely that DNA gain resulted from inter-specific HGT events with mitogenome coding sequences or IGT events with the plastome. The enlarged mitogenomes showed a strong correlation with the presence of sequences with identity to putative nuclear TEs (Additional file 2: Figure S2a). It may be that large mitogenomes are more likely to incorporate DNA from the nuclear genome than small mitogenomes (e.g. the limited transfer window hypothesis) [54, 55]. The TEs may represent remnant tracks of large DNA insertions from the nuclear genome (i.e. MINCs) [20]. To elucidate the evolutionary events of early diverging Fabaceae lineages, more nuclear and mitochondrial reference genomes are needed.

The mitogenome expansion in papilionoid species *Vicia faba* was due to acquisition of large repeat sequences. Several cases similar to the enlargement of *V. faba* [1.4 fold; 407 kb (genome size excluding all but one large repeat) - 588 kb (total genome size)] have been reported within species of various crop plants [e.g., *Beta vulgaris* (368 kb - 501 kb, 1.4-fold) [56]; *Zea mays* (535 kb - 739 kb, 1.4-fold) [57]; *Brassica oleracea* (219–360 kb, 1.6-fold) [58, 59]; and *Oryza sativa* (402 kb - 637 kb, 1.6-fold) [60]. Variation in these species, however, is restricted to specific accessions. An investigation of mitogenomes of *Brassica oleracea* cultivars [61] revealed that genome enlargement occurred by recombination between repeats that are present in all cultivars at low frequency.

In the Papilionoideae, mitogenome size reduction occurred in the ancestor of *Medicago* (Fig. 3). As mentioned above the relatively stable mitogenome size among paraphyletic subsets of Fabaceae does not support a scenario that involves the accumulation of small deletions through time. However, there is evidence that plant mitogenomes can lose large amounts of DNA within a short time period [4, 62, 63]. The mitogenomes of some *Silene* species include dozens of chromosomes and a huge total size of ~ 11 Mb [5, 64]. A recent inter- and intraspecific population level analysis revealed that the mitogenome of *S. noctiflora* passed through a phase of genome reduction driven by the loss of whole chromosomes [63]. Enlarged mitogenomes with more dispersed repeats have a higher probability of forming multipartite mitogenomes, some of which contain chromosomes that are compact and carry no genes. For example, *Leucaena*, which has the largest mitogenome and highest total number of repeats among sampled Fabaceae (Additional file 1: Table S2), includes a putative sub-mitogenomic molecule without genes [39]. Formation of a gene-free mitogenomic chromosome and subsequent exclusion by segregation (e.g. mitochondrial division, cell division or inheritance) could explain compaction of mitogenomes [63]. The combination of mitogenomic chromosome loss, gain of exogenous DNA by IGT and HGT, and acquisition of repetitive DNA likely account for mitogenome size fluctuation in Fabaceae. These processes may explain increases and decreases of mitogenome size across seed plants (Fig. 2).

### Mitochondrial phylogeny and its potential for Fabaceae phylogenetics

The tree topologies from mitochondrial and plastid gene sequences are completely congruent in Fabaceae (Fig. 1). The primary difference between the two trees is in branch lengths, especially in Papilionoideae. The branch length ratio between mitochondrial and plastid trees from non-papilionoids is 1:3.7 compared to 1:7.2 from papilionoids. Plastome-wide nucleotide substitution rate acceleration of papilionoids [65] was attributed in part to shorter generation time because the majority of papilionoid species are herbaceous whereas woodiness is prevalent in the other subfamilies. Mitogenomes of papilionoids also exhibit a slight rate acceleration compared to other subfamilies, which may also be caused by short generation time. The low rate of nucleotide substitution in mitogenome coding regions was suggested to be an advantage for resolving deeper phylogenetic relationships [23, 66]. However, most angiosperm phylogenies are based on plastid and nuclear data while mitogenome data are underutilized [67]. This may be due to the risk of HGT events that cause the incorporation of mitochondrial genes of foreign origin into analyses. This causes conflict in the data and results in unresolved trees and decreased support values for phylogenetic estimation based on multiple genes. Nonetheless, the overall topological congruence and high support values for relationships in both mitogenome and plastome data in Fabaceae suggest coding regions of mitogenomes have not experienced much HGT and that utilizing data from both organelles is potentially useful in future phylogenetic studies of this family.

### Multiple losses of mitochondrial genes and lineage specific intron size variation

Across Fabaceae losses of seven genes were inferred (*cox2*, *rpl2*, *rpl10*, *rps1*, *rps19*, *sdh3* and *sdh4*) (Fig. 4 and Additional file 2: Figure S3). Adams et al. [32] demonstrated multiple losses of several mitochondrial genes across angiosperms using Southern hybridization. The current findings are largely congruent with Adams et al. [32], and the differences are due to the presence of pseudogenes (Additional file 2: Figure S3), some of which likely produced signals in Southern analyses. Three genes (*rpl2*, *rps19*, and *sdh3*) have been lost from Fabaceae mitogenomes multiple times (Fig. 4). Three losses were suggested by the patchy phylogenetic distribution of *sdh3*. While other legumes lack *rpl2* and *rps19* from mitogenomes, all Caesalpinioideae have intact copies. Phylogenetic analysis with other angiosperms (Additional file 2: Figure S4) suggests that the presence of these genes in most legumes is not the result of a recapture event through interspecific HGT but are the remnants of a native ancestral gene.

Shared gene losses from mitogenomes in distinct lineages do not necessarily reflect multiple IGT events because a single ancestral transfer of mitochondrial DNA to the nuclear genome can affect descendant lineages in various ways [24, 25]. The patchy phylogenetic distribution of loss or retention of mitochondrial genes is expected if there is no selective advantage to location in the mitochondrion or nucleus [27]. The example of *rps19* retention in the grass family (Poaceae) [68, 69] is comparable to the retention of *rpl2* and *rps19* in Fabaceae. Several intermediate stages were required for a nuclear copy to silence the mitochondrial gene after IGT [24, 70]. The intermediate steps lasted 60 million years in the case of *rps19* in brome grass (*Bromus inermis*), while deletion of the nuclear copy allowed for retention of the ancestral mitochondrial sequence in rice (*Oryza sativa*) [69]. Meanwhile, other mitochondrial genes in various stages of loss (*rpl10* and *sdh4*) suggest that most gene losses proceed in parallel among the Fabaceae clades (Fig. 4 and Additional file 2: Figure S3).

While gene content variation supports a complex evolutionary history, variation in intron content and length is much simpler, and tends to be restricted to single clades of Fabaceae (Fig. 4). In the case of *cox2*, intron loss is restricted to the Papilionoideae. Intron size variation among three Fabaceae mitochondrial genes (*cox2*, *rps10*, and *ccmFc*) was considerable but not substantially different from other plants. Most land plant mitogenome introns are less than 6 kb [71] but a notable exception is the 11.4 kb *cox2* intron of *Nymphaea* [72]. Several intron elongations have been reported in photosynthetic plant mitogenomes [73]. Large insertions of exotic DNA were a major source of additional intron sequences in *rps10* and *ccmFc* among Fabaceae [37, 40] rather than proliferation of repeat sequences as reported from other land plants [e.g. *Psilotum* (fern ally) [71]; *Cycas* (gymnosperm) [74]; and *Nymphaea* (basal angiosperm) [72]]. Chang et al. [37] suggested that a mitovirus was a putative source of additional intron sequences of *rps10*, and the origin of additional intron sequence of *ccmFc* remains unknown [40].

Nucleotide alignment of the *cox2* gene from *Leucaena* and other legumes suggested a sequence insertion (ca. 2.9 kb) into the intron (Additional file 2: Figure S5). Although BLAST analyses using the unique intron sequence to query the NCBI database did not produce strong matches, large portions (~ 82.8%) had high nucleotide identity (94.9%) to an IGS region of the caesalpinioid genus *Haematoxylum* (Additional file 2: Figure S5). This suggests that the shorter *cox2* intron of *Haematoxylum* and *Libidibia* represents the ancestral length in the clade including *Leucaena*. The insertion of *nad4L* gene within *nad1* intron was recognized from *Selaginella* (fern ally) [75]. The *cox2* intron elongation shown from Caesalpinioideae however, is likely due to a transfer of IGS sequence into the intron, a phenomenon that has not yet been fully appreciated in mitogenomes of seed plants.

### Decay of mitogenomes with genetic distance

All pair-wise relationships among 12 Fabaceae species agree with the findings of Guo et al. [53] for selected clades of seed plants, showing a strong negative correlation between the amount of shared DNA and coding region sequence divergence (Additional file 1: Table S3, S4; Fig. 5). One notable finding is that the half-life, when 50% of two mitogenome sequences are no longer shared, is very short. This supports the suggestion that most mitogenome DNA is likely not functional [76]. However, for the most part each Fabaceae species, including the most divergent taxa, share at least 100 kb of sequence comprising genic regions and IGS in close proximity to genes. These regions represent the core of the mitogenome, and this value is comparable to the amount of shared DNA in Asteraceae mitogenomes (ca. 88 kb) [77]. Christensen [2, 6] hypothesized that the combination of accurate and error-prone double strand break repair mechanisms and subsequent selection on mitogenomic molecules in the absence of deleterious mutation in coding regions can produce this phenomenon. However, the detailed mechanisms, the contribution of foreign sequence migration (i.e. interspecific HGT) and pre-existing sequence variation for rapid IGS change [78] remain obscure. Whatever caused the rapid decline in shared mitogenomic DNA, there is clear evidence of conservation of coding sequences in Fabaceae (Fig. 5).

### Horizontal transfer from mimosoid species to *Lophophytum*

Analyses of four new mitogenomes from Cercidoideae, Detarioideae and two non-mimosoid Caesalpinioideae (Fig. 6) enhance support for extensive horizontal mitogenome transfer from mimosoid legumes to *Lophophytum* [39, 42]. However, two important questions remain: 1) Is the mimosoid host species the only contributor to *Lophophytum* mitogenome among the legumes [39] and 2) how much DNA was transferred from legumes to *Lophophytum*? [42] Comparing the amount *Lophophytum* (Fig. 6a) and *Leucaena* (Fig. 6b) DNA shared across 11 legume mitogenomes showed an identical pattern, ruling out the possibility that the massive amount of foreign DNA in *Lophophytum* originated from non-mimosoid Fabaceae. The extent of DNA shared between *Leucaena* and *Lophophytum* (~ 330 kb) suggests that the shared DNA of the actual mimosoid host species (e.g. *Anadenanthera*, *Enterolobium*, *Inga*, *Piptadenia*, *Pithecolobium*) [79] is higher than 330 kb (Additional file 2: Figure S6). Moreover, the consistent percentage of shared DNA (average of 87.5%) between two analyses (Fig. 6c) may indicate that *Lophophytum* contains at least 87.5% DNA of the host mimosoid mitogenome. The fact that the first half-life period of mitogenome decay is short even among very closely related genera (Fig. 5) suggests that this estimate is conservative.

## Conclusions

The complex structure of plant mitogenomes has made it challenging to perform comparative analyses due to the paucity of complete sequences. During the past decade as more mitogenome sequences became available the understanding of the patterns and causes of the bizarre variation has greatly improved, however, there are very few clades for which multiple mitogenome sequences are available for comparative analyses. One family that has been a focus of mitogenome comparisons is Fabaceae but most of these investigations were based on species from one of the six recognized subfamilies, Papilionoideae. The four newly sequenced mitogenomes reported here extend the phylogenetic coverage to four subfamilies. The family has experienced several substantial mitogenome size fluctuations in both ancient and recent times. The causes of these size variations are distinct in different lineages. Multiple, independent losses of seven genes occurred throughout the evolutionary history of Fabaceae. In contrast, variation in intron content and length is restricted to single clades. Finally, the expanded sampling of lineages across the Fabaceae provides new insights into transfer of mitogenome sequences from Fabaceae into the parasitic plant *Lophophytum*.

## Methods

### Mitogenome assembly and validation

Four species of Fabaceae, *Cercis canadensis*, *Tamarindus indica, Libidibia coriaria* (= *Caesalpinia coriaria*) and *Haematoxylum brasiletto*, were selected for the mitogenome sequencing. The seeds of four species were originally obtained from eBay (*L. coriaria*) and USDA-ARS National Plant Germplasm System (*C. canadensis*, *T. indica,* and *H. brasiletto*) in Schwarz et al. [80]. Voucher specimens were identified by Erika N. Schwarz and deposited to University of Texas at Austin herbarium (TEX-LL). The raw reads generated by Schwarz et al. [80] were used for mitogenome assembly. These 100 bp paired end reads were generated by Illumina (San Diego, CA) sequencing of libraries containing inserts of ca. 700 bp. Genome assembly and read mapping were conducted by the Geneious mapper and aligner, respectively, in Geneious 7.1.9 (https://www.geneious.com). Prior to mitogenome assembly, plastome reads were identified and excluded by mapping all reads to the corresponding complete plastomes [*C. canadensis* (KF856619), *T. indica* (KJ468103), *L. coriaria* (KJ468095) and *H. brasiletto* (KJ468097)] using custom options (1% maximum each gaps and mismatches allowed, only map paired reads that match nearby, save list of unused reads). De novo assembly was conducted with low sensitivity in Geneious using approximately 30,000,000 plastome-filtered reads. All plastome-filtered reads were then mapped against assembled contigs. Putative nuclear genome contigs with low coverage (< 100X) were excluded. Among the remaining contigs, high coverage nuclear contigs (e.g. ribosomal DNA repeat unit) were excluded and mitochondrial contigs were selected by BLAST searches against reference Fabaceae mitogenome sequences at NCBI (https://www.ncbi.nlm.nih.gov/genome/organelle/) using BLASTN 2.8.0+ [81] with default options.

To complete and validate mitogenome assemblies, polymerase chain reactions (PCRs) were performed with primers designed in Primer3 [82]. Primer sequences and target regions are listed in Additional file 1: Table S5. Gaps between contigs, dispersed repeats, MIPTs larger than the ca. 700 bp library inserts, and junctions between large repeats and single copy regions were confirmed by PCR and Sanger sequencing. For MIPTs larger than 1.5 kb, long-range PCR was performed with TaKaRa PrimeSTAR GXL (Takara Bio USA, Inc., Mountain View, CA, USA) and Sanger sequencing of the amplicons was completed by nested PCR at the University of Texas Genomic Sequencing and Analysis Facility in Austin. Manual refinement of each mitogenome was conducted in Geneious. Finally, coverage for assembled and refined mitogenomes was checked by two different datasets and options. The first mapping used plastome-filtered reads with custom options (5% maximum each gaps and mismatches allowed, only map paired reads that match nearby). The second mapping used total raw single end reads with low sensitivity option.

### Genome annotation and analysis

In addition to the four completed mitogenomes, eight previously published mitogenomes were reannotated (Additional file 1: Table S6) for comparative analyses. Annotation of Fabaceae mitogenomes utilized Geseq [83]. Three mitogenomes were selected (*Psilotum nudum*: KX171638 and KX171639; *Ginkgo biloba*: KM672373; *Liriodendron tulipifera*: NC_021152) as BLAT reference sequences, each of which contained a set of 41 conserved, ancestral seed plant protein-coding genes [53, 71, 84]. Annotations were evaluated and manually corrected in Geneious. The start and stop codons were manually adjusted to fit open reading frames. Exon and intron boundaries were determined by comparisons to conserved syntenic regions of other published mitogenomes retrieved from NCBI (https://www.ncbi.nlm.nih.gov/genome/organelle/). The tRNAs were also checked by tRNAscan-SE v2.0 [85]. All four completed and annotated mitogenomes were deposited in GenBank (MN017226 - MN017229).

Putative transposable elements (TEs) were investigated using the CENSOR webserver [86] with default parameters and Viridiplantae was selected as a sequence source, as described by Park et al. [87]. The number and length of MIPTs was evaluated by BLASTN 2.8.0+ with the default option. Each mitogenome was used as the query against a subject database comprising the corresponding plastome (Additional file 1: Table S6). Putative MIPTs less than 100 bp were excluded from the estimation.

The distribution of two kinds of repeats (tandem and dispersed) was examined in each mitogenome. Tandem repeats were analyzed using Tandem Repeats Finder version 4.09 [88] with default options. Dispersed repeats were identified by using each mitogenome as both subject and query in BLASTN analysis with a word size of 7 and an e-value of 1e-6 following Guo et al. [53], enabling detection of repeats as short as 30 bp. All blast hits were retained. Sequence coordinate information for tandem and dispersed repeats was transferred to each mitogenome as an annotation in Geneious. Overlapping regions between repeats were excluded from the estimations and the percentage of repetitive and single copy DNA in the mitogenomes was calculated. The number of BLAST hits for repeats was estimated for four size intervals (30–100, 101–300, 301–1000 and > 1001 bp). In estimating the total number of repeats, partly or wholly overlapping repeats were treated as a single repeat unit.

### Estimation of mitogenome size variation in seed plants

To estimate size variation in mitogenomes of seed plants, 106 available complete mitogenomes from NCBI were evaluated (https://www.ncbi.nlm.nih.gov/genome/organelle/; accessed on Oct. 16, 2018).

### Phylogenetic analysis

To infer phylogenetic relationships among mitogenomes of Fabaceae and test congruence with the plastome phylogeny, maximum likelihood (ML) analyses were conducted on data from both genomes. In addition to 12 representative Fabaceae species, taxon sampling included two other genera of the nitrogen-fixing clade (*Cucurbita* and *Rosa*) and two genera of Malphigiales (*Populus* and *Salix*) as outgroups (Additional file 1: Table S6). All shared mitochondrial (26) and plastid (68) protein coding genes from 16 taxa were extracted from each organelle genome (Additional file 1: Table S7). Gene sequences from each organelle genome were concatenated in two separate data sets and aligned with MAFFT v.7.017 [89] using default options. Poorly aligned regions were deleted or manually adjusted for each alignment. Nucleotide substitution models were selected by Akaike information criterion (AIC) in jModelTest v.2.1.6 [90]. ML analysis (GTR + I + G with 1000 bootstrap replications) was conducted using RAxML v.8 [91] in the CIPRES Science Gateway [92].

To test the phylogenetic position of *rpl2* and *rps19* genes of four Caesalpinioideae mitogenomes, sequences of representative angiosperms were extracted from published mitochondrial sequences (Additional file 1: Table S6). *Cercis canadensis* from the Cercidoideae was also included in this analysis. Alignment and ML analyses (GTR + G) were performed as described above.

### Shared DNA analysis

Shared DNA among the 12 Fabaceae mitogenomes was estimated by pairwise comparisons using BLASTN with the same parameters as the repeat analyses. To avoid overestimation of shared DNA by including large repeats, all but one copy of repeats > 1 kb was manually deleted from all mitogenomes. Pair-wise Kimura 2-parameter (K2P) [93] genetic distance was calculated for 12 Fabaceae species using MEGA version 7.0 [94] based on the alignment of the 26 mitochondrial CDS sequences employed for the phylogenetic analysis.

Shared DNA was also estimated to detect HGT using concatenated sequences of the multipartite mitogenomes (KU992322–KU992380 and KX792461) of the holoparasitic *Lophophytum* (Balanophoraceae) as a query and 12 Fabaceae mitogenomes as a subject. A similar comparison was performed with *Leucaena* as a query and the 12 species (11 other Fabaceae species and *Lophophytum*) as the subject. The percentage of shared DNA between the comparisons was calculated by dividing the values for *Lophophytum* by the values for *Leucaena*.

## Supplementary information

**Additional file 1: Table S1.** Assembly information of four completed Fabaceae mitogenomes. **Table S2.** Summary of repeat sequences in mitogenomes of Fabaceae. **Table S3.** Pairwise comparison of shared DNA (kb) among the mitochondrial genome of Fabaceae species. **Table S4.** Pairwise K2P distance among the mitochondrial genome of Fabaceae species. **Table S5.** Primer sequences and resulting amplification products from mitogenome finishing. **Table S6.** Accession numbers of mitogenomes used in comparative genomics and phylogenetic analyses. **Table S7.** Mitochondrial and plastid genes included in phylogeny reconstructions.

**Additional file 2: Figure S1.** Read coverage of four complete Fabaceae mitogenomes. **Figure S2.** Factors contributing to mitogenome size in 12 representative Fabaceae. **Figure S3.** Gene content of the 12 Fabaceae mitogenomes. **Figure S4.** Maximum likelihood phylogenies for *rpl2* and *rps19*. **Figure S5.** Variation in the *cox2* gene among Fabaceae mitogenomes. **Figure S6.** Schematic relationships of shared mitochondrial DNA between Fabaceae and holoparasitic *Lophophytum* (Balanophoraceae).

## Data Availability

All four completed and annotated mitogenomes were deposited in GenBank (MN017226 - MN017229).

## Abbreviations

bp: Basepairs
CDS: Coding sequence
HGT: Horizontal gene transfer
IGS: Intergenic spacer
IGT: Intracellular gene transfer
IQR: Interquartile range
K2P: Kimura 2-parameter
kb: Kilobasepairs
MINC: Mitochondrial DNA of nuclear origin
MIPT: Mitochondrial DNA of plastid origin
ML: Maximum likelihood
PCR: Polymerase chain reaction
TE: Transposable element
