# Supplementary material for: Fluctuations in Fabaceae mitochondrial genome size and content are both ancient and recent
Source: BMC Plant Biol. 2019 Oct 25;19:448. doi: 10.1186/s12870-019-2064-8 (PMC6814987; doi:10.1186/s12870-019-2064-8)
Supplement: Supplementary file 2 — Additional file 2: Figure S1. Read coverage of four complete Fabaceae mitogenomes. Figure S2. Factors contributing to mitogenome size in 12 representative Fabaceae. Figure S3. Gene content of the 12 Fabaceae mitogenomes. Figure S4. Maximum likelihood phylogenies for rpl2 and rps19. Figure S5. Variation in the cox2 gene among Fabaceae mitogenomes. Figure S6. Schematic relationships of shared mitochondrial DNA between Fabaceae and holoparasitic Lophophytum (Balanophoraceae). [file 12870_2019_2064_MOESM2_ESM.docx]

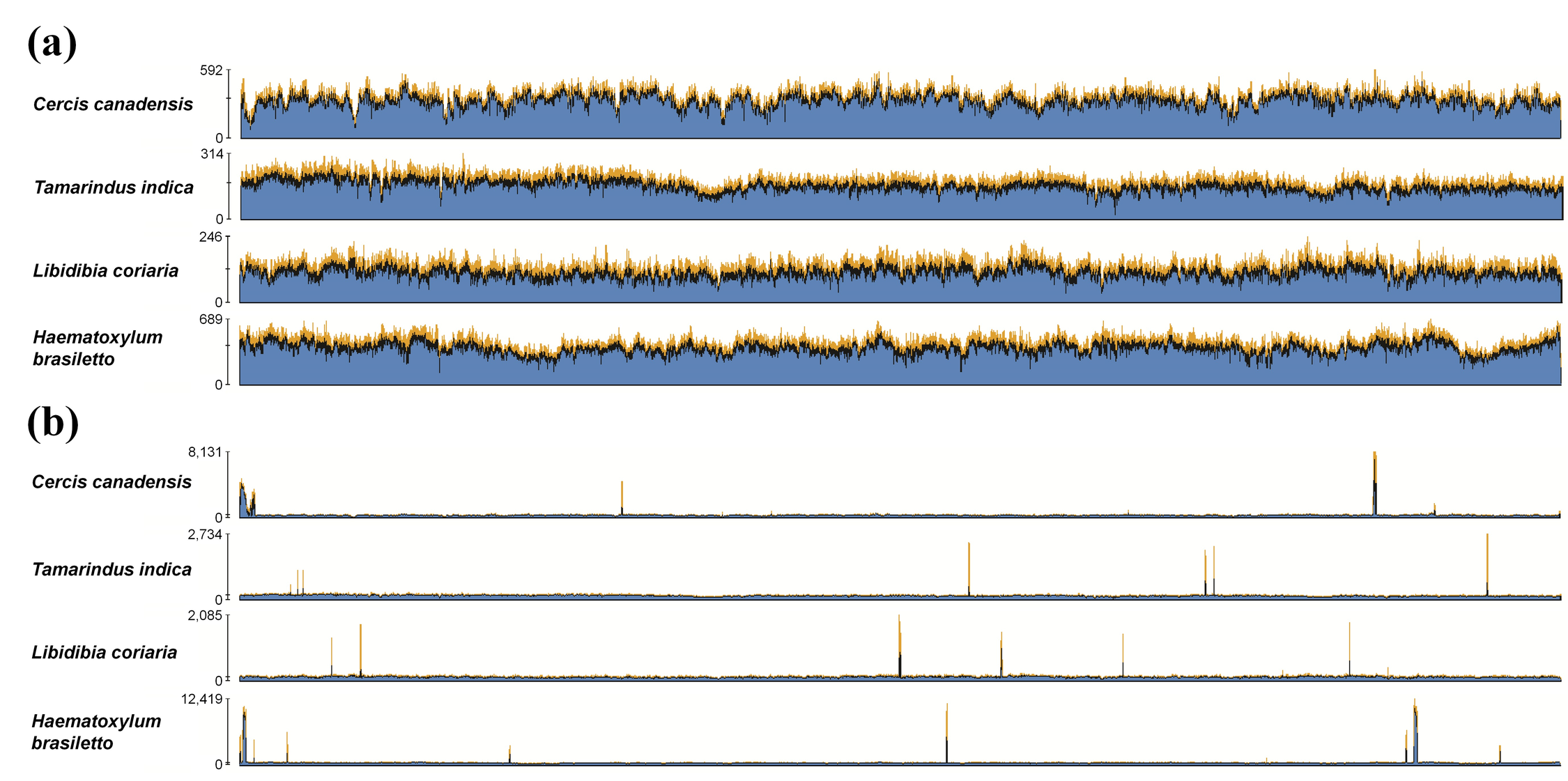


**Fig. S1** Read coverage of four complete Fabaceae mitogenomes. (a) Coverage with plastome filtered reads using paired end data. (b) Coverage with total reads without paired end data. The mitochondrial regions with high coverage (peaks) represent sequences that originated from plastome (MIPTs). Scale on left of each graph indicates coverage, which is represented by the histogram (blue = minimum, black = mean, yellow = maximum). The horizontal axis represents the position of each assembled genome and lengths are not proportional for each species.


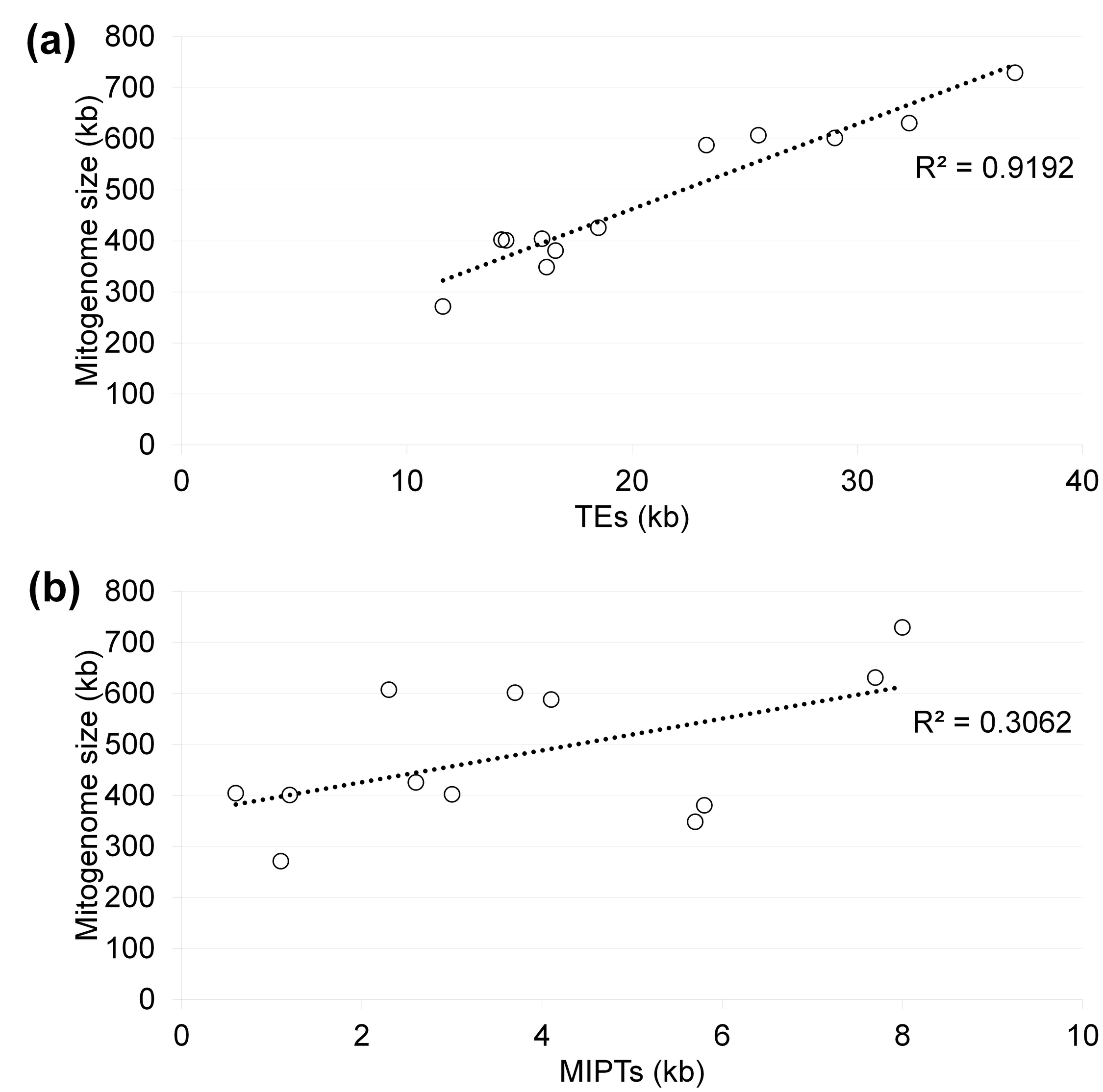


**Fig. S2** Factors contributing to mitogenome size in 12 representative Fabaceae. (a) Correlation between mitogenome size and the total length of transposable elements (TEs). (b) Correlation between mitogenome size and total length of mitochondrial DNA of plastid origin (MIPTs). Open circles in the plots represent the 12 Fabaceae included in the analysis.


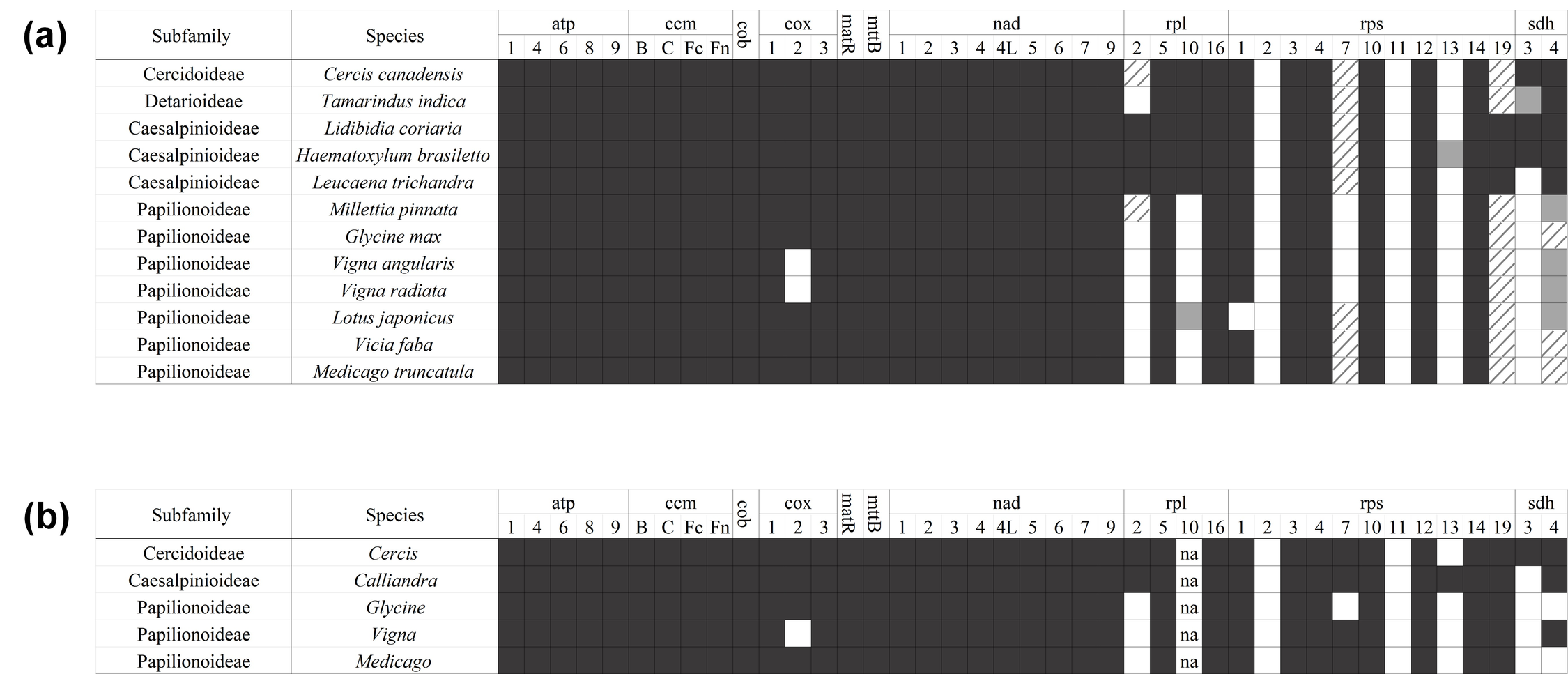


**Fig. S3** Gene content of the 12 Fabaceae mitogenomes. (a) Twelve mitogenomes examined in this study. (b) Five Fabaceae species examined in Adams et al. (2002) using Southern hybridization methods. Black = present, gray = putative pseudogene, diagonal lines = truncated gene, white = absent, na = not applicable. If several genes occur within a gene group/family, the type of gene or gene family abbreviation is presented in the first row and the specific gene is indicated in the second row.


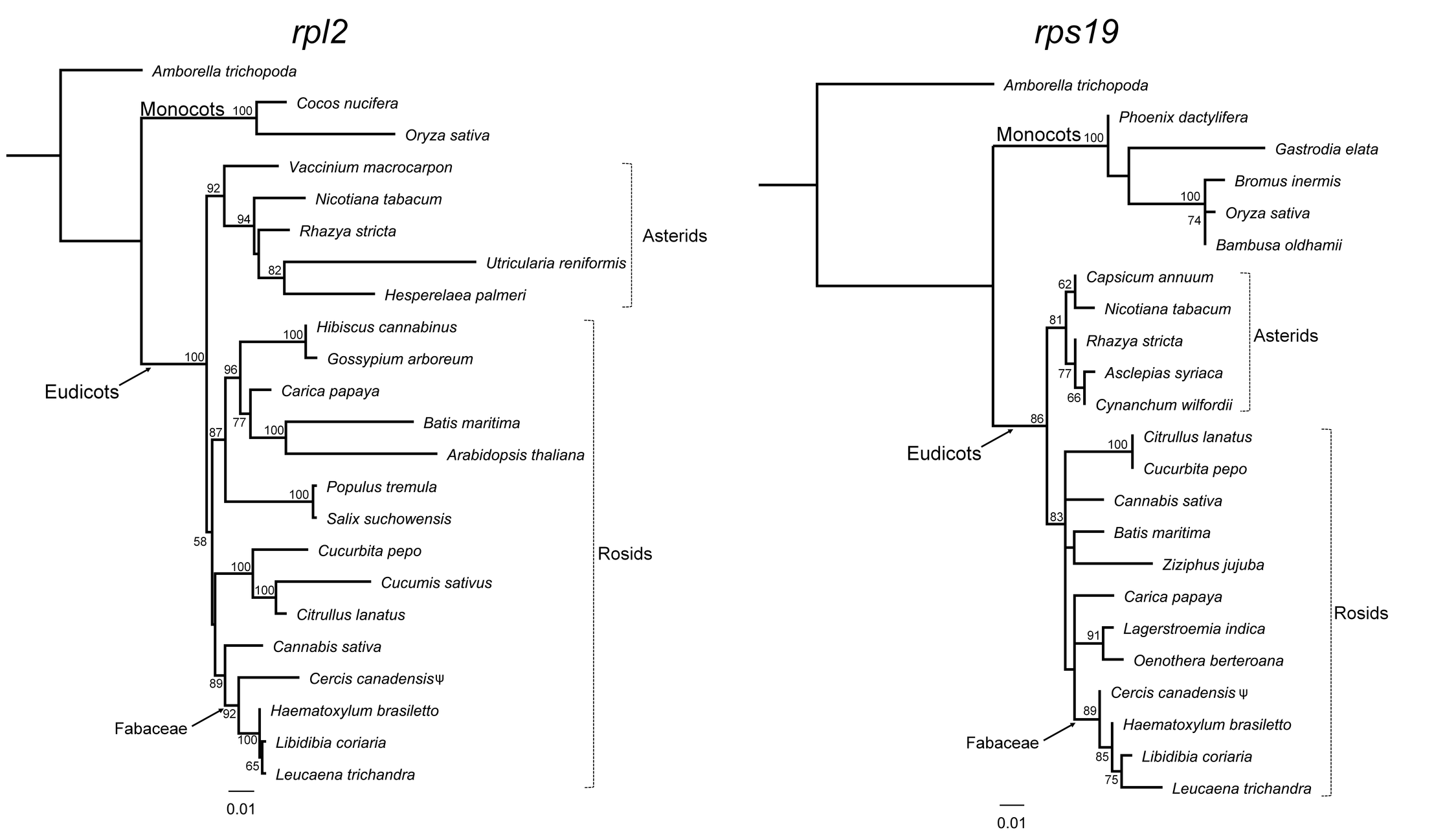


**Fig. S4** Maximum likelihood phylogenies for *rpl2* and *rps19*. Bootstrap values > 50% are indicated at nodes. Psi (ψ) indicates a truncated copy of the gene in a given taxon. NCBI accession numbers are listed in Table S6. Scale indicates number of nucleotide substitutions per site.


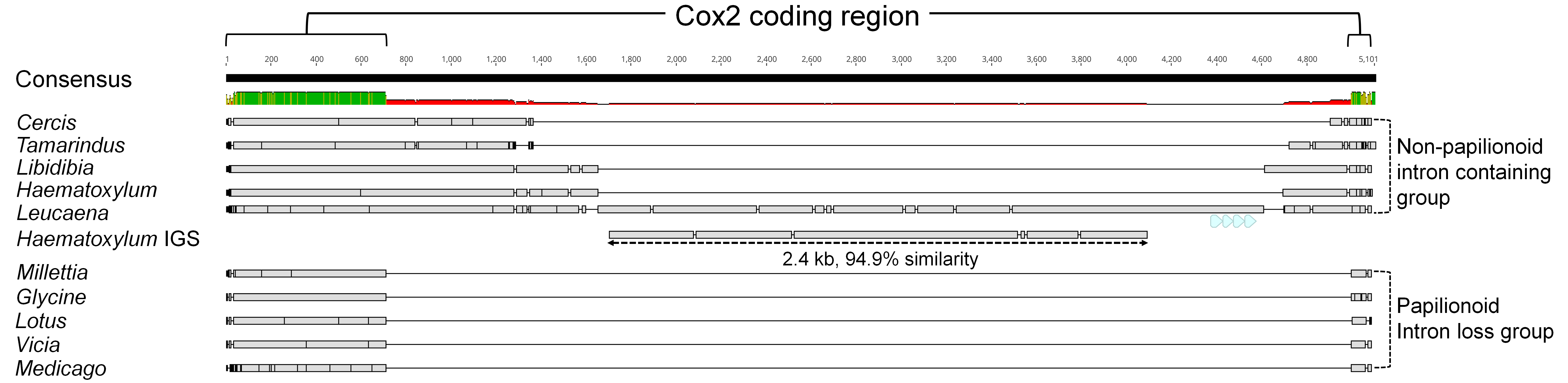


**Fig. S5** Variation in the *cox2* gene among Fabaceae mitogenomes. The *cox2* gene of 10 species and intergenic spacer (IGS) of *Haematoxylum* are included. The alignment was manually adjusted to minimize gaps and maximize apparent homologous regions. The majority consensus was determined with 0% threshold and without gaps. Nucleotide coordinates within the alignment are indicated on thick, horizontal black line; consensus sequence identity is shown below coordinates (green = 100% identity, yellow-green = at least 30% and under 100% identity, red = below 30% identity). An array of sky-blue pentagons represents tandem repeat.


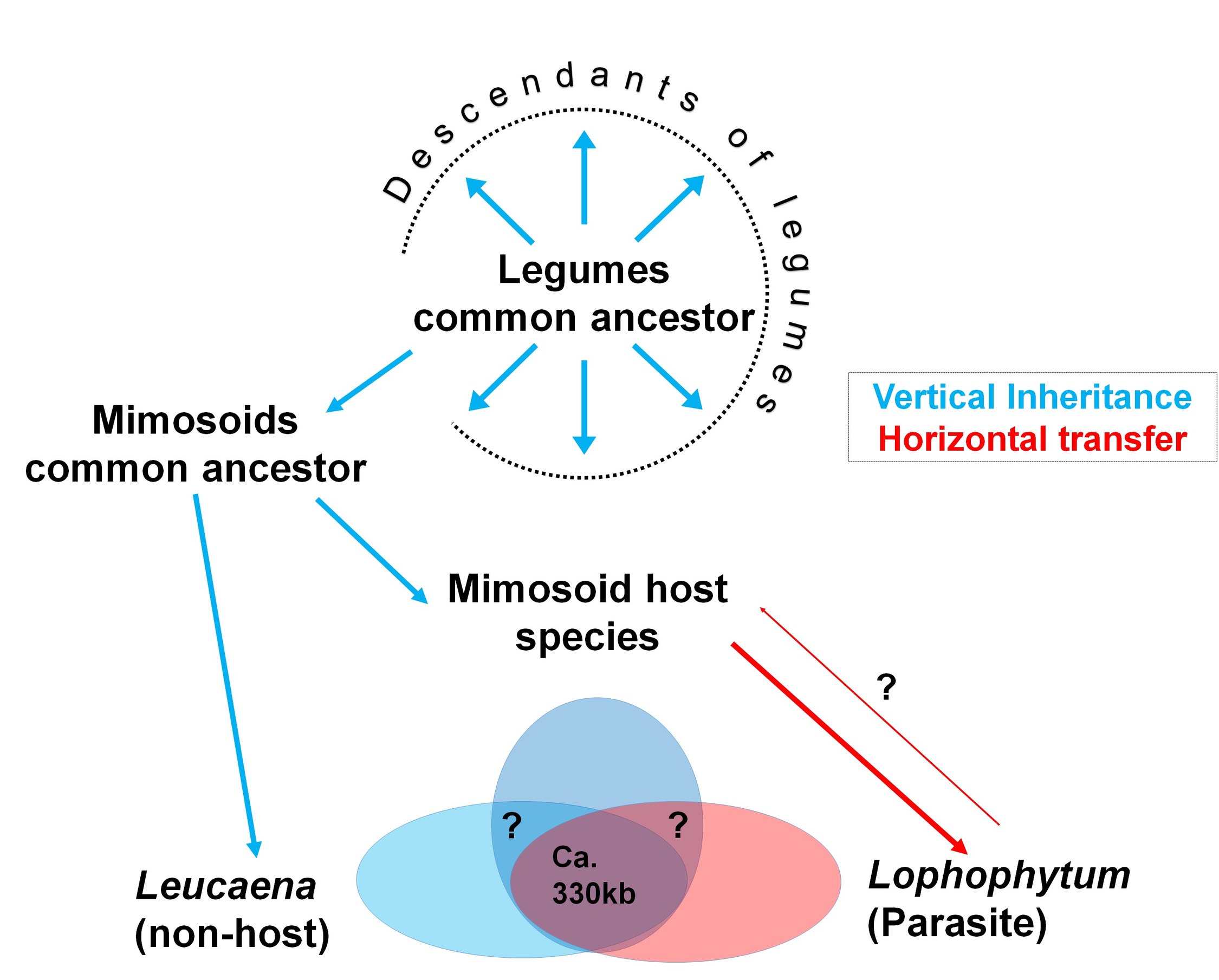


**Fig. S6** Schematic relationships of shared mitochondrial DNA between Fabaceae and holoparasitic *Lophophytum* (Balanophoraceae).
